# Footwear and insole design features that reduce neuropathic plantar forefoot ulcer risk in people with diabetes: a systematic literature review

**DOI:** 10.1186/s13047-020-00400-4

**Published:** 2020-06-04

**Authors:** Sayed Ahmed, Alex Barwick, Paul Butterworth, Susan Nancarrow

**Affiliations:** School of Health and Human Sciences, Southern Cross University, Billinga, Queensland 4225 Australia

**Keywords:** Diabetic foot, Footwear, Insoles, Plantar pressure

## Abstract

**Background:**

In people with diabetes, offloading high-risk foot regions by optimising footwear, or insoles, may prevent ulceration. This systematic review aimed to summarise and evaluate the evidence for footwear and insole features that reduce pathological plantar pressures and the occurrence of diabetic neuropathy ulceration at the plantar forefoot in people with diabetic neuropathy.

**Methods:**

Six electronic databases (Medline, Cinahl, Amed, Proquest, Scopus, Academic Search Premier) were searched in July 2019. The search period was from 1987 to July 2019. Articles, in English, using footwear or insoles as interventions in patients with diabetic neuropathy were reviewed. Any study design was eligible for inclusion except systematic literature reviews and case reports. Search terms were diabetic foot, physiopathology, foot deformities, neuropath*, footwear, orthoses, shoe, footwear prescription, insole, sock*, ulcer prevention, offloading, foot ulcer, plantar pressure.

**Results:**

Twenty-five studies were reviewed. The included articles used repeated measure (*n* = 12), case-control (*n* = 3), prospective cohort (*n* = 2), randomised crossover (n = 1), and randomised controlled trial (RCT) (*n* = 7) designs. This involved a total of 2063 participants. Eleven studies investigated footwear, and 14 studies investigated insoles as an intervention. Six studies investigated ulcer recurrence; no study investigated the first occurrence of ulceration. The most commonly examined outcome measures were peak plantar pressure, pressure-time integral and total contact area. Methodological quality varied. Strong evidence existed for rocker soles to reduce peak plantar pressure. Moderate evidence existed for custom insoles to offload forefoot plantar pressure. There was weak evidence that insole contact area influenced plantar pressure.

**Conclusion:**

Rocker soles, custom-made insoles with metatarsal additions and a high degree of contact between the insole and foot reduce plantar pressures in a manner that may reduce ulcer occurrence. Most studies rely on reduction in plantar pressure measures as an outcome, rather than the occurrence of ulceration. There is limited evidence to inform footwear and insole interventions and prescription in this population. Further high-quality studies in this field are required.

## Background

Foot ulcers are a common consequence of diabetes due to the development of peripheral neuropathy, peripheral vascular disease, limited joint mobility and foot deformity [1–6]. Nearly 34% of persons with diabetes will develop a foot ulcer in their lifetime [7]. This can lead to infection and amputation; diabetes is the main reason for non-traumatic lower limb amputation [8, 9]. Previous foot ulcer or amputation is a risk of future amputation [1, 3, 5, 10]. Additional risk factors include higher Body Mass Index (BMI), and structural foot deformities [2–4, 6], such as hammertoes and hallux valgus [11, 12].

Diabetic peripheral neuropathy (DPN) is the central risk factor for the development of plantar foot ulceration [13]. Over 30% of persons with diabetes will develop DPN [14], the incidence increasing with age [15, 16]. DPN can affect the autonomic, sensory and motor nervous systems. Sensory neuropathy interrupts the protective feedback mechanism of touch and pain [17]. Motor neuropathy results in compromised muscle innervation, reduction in strength, and combined with limited joint mobility, the development of foot deformities. These deformities may lead to an increase in plantar foot pressures, particularly in the forefoot [18–21]. Autonomic neuropathy leads to diminished sweating and changes to skin perfusion, leading to dry skin and hyperkeratosis. As skin integrity is compromised, patients are more susceptible to trauma which may precipitate a diabetic foot ulcer [21–24].

Neuropathic ulcers in diabetic feet occur mostly at the plantar forefoot [11, 25, 26] and correspond to areas of peak plantar pressure [27]. Bennetts et al. [28] demonstrated that most peak pressure areas are located in the forefoot regions in this population. Limited range of motion at the forefoot joints is also likely to contribute to the peak plantar pressures (PPP) observed in this region [29]. For this reason, plantar pressure mapping is used to guide footwear and insole manufacture and judge their effectiveness [30].

Reducing plantar pressures is considered a key factor for wound healing and prevention of ulcer recurrence [31, 32]. Footwear and insoles are an essential treatment modality for offloading these pressures [33, 34]. The desired offloading threshold should be > 30% reduction in dynamic in-shoe plantar pressure from the baseline or < 200 kPa to ensure ulcer-free survival at the forefoot [35]. This systematic review aimed to summarise and evaluate the evidence for footwear and insole features that reduce pathological plantar pressures and the occurrence of diabetic neuropathy ulceration at the plantar forefoot in people with diabetic neuropathy.

## Methods

The systematic search was performed according to the Preferred Reporting Items for Systematic Reviews and Meta-Analysis (PRISMA) Statement [36].

### Search strategy

**Fig. 1:**
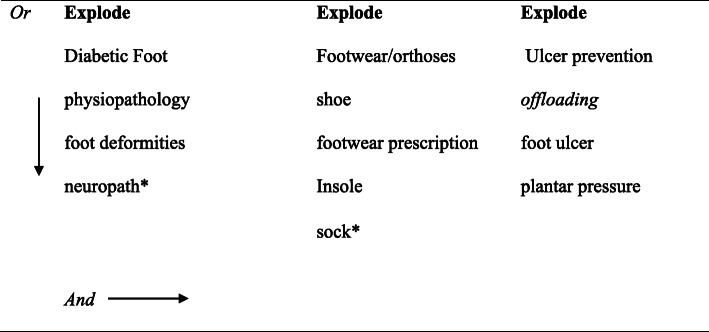
Search terms used to select the studies

In July 2019, six electronic databases were searched (Medline, Cinahl, Amed, Proquest, Scopus, Academic Search Premier) using medical subject headings followed by a keyword subject heading. The search period was from 1987 to July 2019. The search terms can be seen in Fig. 1 and Supplementary file 1.

### Eligibility criteria

All studies included in the systematic review were obtained from full-text peer-reviewed journals published in English. Studies that did not use footwear or insole as a mode of intervention for long term offloading were excluded. Letters to the editor, opinion pieces, conference proceedings, and editorials were also excluded. All study designs except systematic reviews and case reports were eligible for inclusion. The titles and abstracts of the articles were screened by one reviewer (SA). Full-text articles were reviewed based on the following criteria: i, participants were adult (> 18 years), had diabetes; ii, all or some of the participants had neuropathy and foot deformity, history of plantar forefoot ulcers but no Charcot foot, history of heel ulcer or active foot ulcers; iii, studies used footwear or insoles as a long-term offloading intervention; iv, the outcome of the study was either (re)occurrence of forefoot ulcer or change in forefoot plantar pressure outcomes; v, the footwear or insole interventions had to be sufficiently described to be able to draw useful conclusions; vi, conventional materials and manufacturing techniques were used; and vii, closed-in footwear was used. The reference lists of studies obtained through the database search were also searched to identify relevant citations.

### Quality assessment

Quality assessment was performed independently by two reviewers (SA and AB). The quality assessment form was adapted from the McMaster Critical Review Form – Quantitative Studies [37].

## Results

**Fig. 2:**
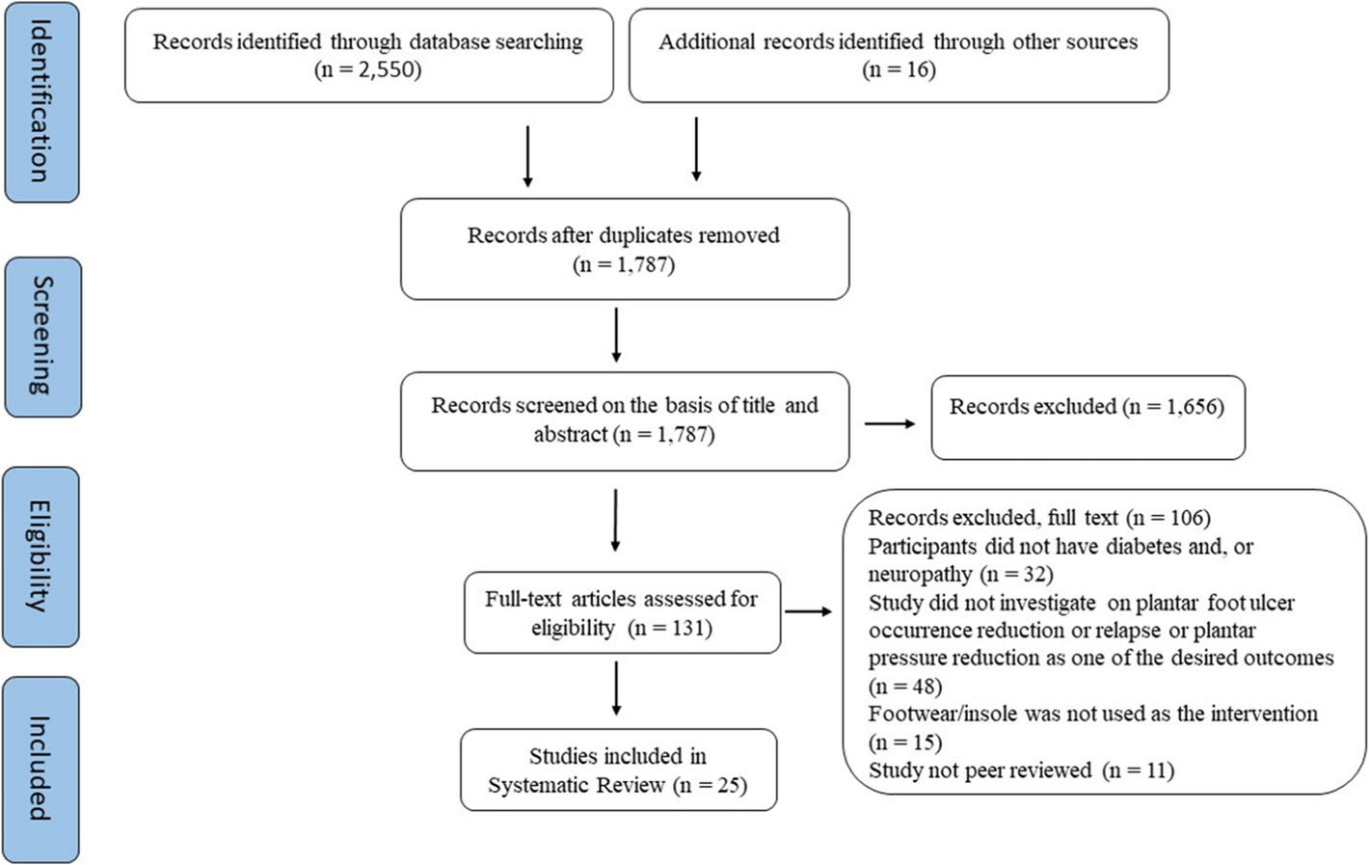
PRISMA Study Selection Flow Diagram

The literature search identified 1787 articles. Twenty-five articles met the eligibility criteria to be included in the review (Fig. 2). The study designs included repeated measures (*n* = 12), case-control (*n* = 3), prospective cohort (*n* = 2), randomised crossover (n = 1), and RCT (*n* = 7) studies.

Study characteristics are shown in Tables 1 and 2.

**Table 1:** Characteristics of the selected studies that used pressure reduction as the primary outcome measure

Author, date	Location	Study design	Follow up period	Sample size	Sample characteristics	Intervention & Comparison	Outcome measures	Result
Arts et al. 2012 [38]	Netherlands	Repeated measures	Same day	171 (336 ft)	Diabetic neuropathy Previous plantar ulcer	Custom-made footwear Semi-customised footwear Barefoot	Peak plantar pressure (PPP) of < 200 kPa considered successful	Custom-made footwear is least effective in pressure reduction (< 200 KPa) at forefoot compared to midfoot and known ulcer locations (29% vs 81 and 62%)
Arts et al. 2015 [39]	Netherlands	Repeated measures	Same day	85	Diabetic neuropathy Previous plantar foot ulcer	Various footwear modifications to custom or semi-custom footwear Footwear before modification	% plantar pressure reduction	MP, local cushion and plastazote top cover reduce PP respectively by15.9, 15, 14.2% and combinedly 24 and 22% at the forefoot.
Bus et al. 2011 [30]	Netherlands	Repeated measures	Not reported	23	Diabetic Neuropathy, Foot deformity Foot ulcer	Fully custom-made footwear and insoles	In-shoe plantar pressure reduction by more than 25% (Criteria A) or below the absolute value of 200 kPa (Criteria B)	MB or MP, replacing the top cover, early rocker can reduce pressure at hallux and metatarsal area ranging from 10.1 to18.6% as an individual modification.
Bus et al. 2004 [40]	Netherlands	Repeated measure	Not reported	20	Diabetic Neuropathy, History of healed plantar foot ulcers Foot deformity	Insoles; 9.5 mm thick flat PPT insole and custom-made insoles out of open-cell urethane foams of hardness 60–80. Custom-made insoles were made by CADCAM process.	Plantar pressure reduction FTI	Custom-made insoles reduce plantar pressure and FTI significantly at medial and lateral heal, MTH1 and FTI at lateral MTHs when compared with flat PPT insoles.
Charanya et al. 2004 [41]	India	Case-control study	6 months	25	Diabetic Neuropathy History of active and healed plantar ulcers Non-diabetic (Control)	Footwear with an insole made of 12 mm MCR, shore value 20^0^, Toughened rocker profile rubber outsole	Foot sole hardness reduced close to normal, shore value 20^0^	Plantar ulcers healed in three-four weeks, foot sole skin hardness reduced to 25–30 from 45 to 50 shore values.
Guldemond et al. 2007 [42, 43]	Netherlands	Repeated measures	Not reported	17	Diabetic Neuropathy Higher barefoot plantar pressure (≥700 kPa)	Insole with various height arch supports and with and without a metatarsal dome	In-shoe plantar pressure reduction (36% & 39%), Walking convenience on a 10-point rating scale	Extra arch support and MD are respectively effective in 39% & 36% pressure reduction in central and medial regions of the forefoot
Hastings et al. 2007 [44]	USA	Repeated measure	22 months	20	Diabetic Neuropathy History of plantar foot ulcers No active foot ulcers No Charcot neuropathy	Three footwear conditions; extra depth footwear with 1) Total Contact Insoles (TCI), 2) TCI with proximal Metatarsal Pad (MP), 3) TCI with distal MP, CT Scan	PPP CT Scan for positioning of MP against MTHs	Highest (57%) PPP reduction occurred at 2nd MTH when MP placed at 10.6 mm proximal to MTH line. Variable PPP under the 2nd MTH varied between 32 ± 16% when positioning of MP varies between 6.1 mm to 10.6 mm proximal to MTH line.
Lin et al. 2013 [45]	China	Repeated measure	Not reported	26	Diabetic Neuropathy	Insole with pre-plug removal, post-plug removal, and post-plug removal + arch support	Mean peak pressure (MPP), maximum force, contact area	Removing insole plug is effective in offloading MPP by 32.3% and adding arch support reduces further 9.5% at the forefoot
Lott et al. 2006 [46]	USA	Repeated measure	Not reported	20	Diabetic Neuropathy History of midfoot or forefoot plantar ulcers	Four different conditions; 1) Barefoot, 2) Footwear, 3) Footwear + TCI, 4) Footwear + TCI + MP	Plantar pressure reduction Soft tissue thickness (STT)	PP & ST strain under 2nd MTH are highest at the barefoot condition and lowest at footwear + TCI + MP condition. Mean PP for all four conditions under 2nd MTH is 272 kPa, 173 kPa, 140 kPa and 98 kPa.
Martinez-Santos et al. 2019 [47]	UK	Repeated measure	Not reported	60	Diabetic Neuropathy No previous ulcers	Insole with three different metatarsal bar (MB) positioning, two different types of materials	PPP	Maximum pressure reduction can be achieved by positioning metatarsal bar at 72% length of insole, irrespective of material type
Mueller et al. 2006 [48]	USA	Repeated measure	Not reported	20	Diabetic Neuropathy history of plantar ulcers	Three footwear conditions: 1) Footwear, 2) Footwear with TCI, and 3) Footwear with TCI + MP	PPP PTI STT	TCI and metatarsal pad caused reductions of pressure under the metatarsal heads
Owings et al. 2008 [49]	USA	Repeated measure	Not reported	20	Diabetic Neuropathy Higher (> 750 kPa) barefoot plantar pressure at MTH region	Three different type custom-made insoles (X, Y from shape-based and Z combined foot shape with plantar pressure data). Footwear with rigid rocker sole and flexible sole	Peak pressure FTI	Shape and pressure-based insoles (Z) showed improved offloading by 32 and 21%, PTI reduction 40 and 34% when compared to shape-only-based insoles (X-Polypropylene base, Y- EVA base). A similar trend was observed in flexible and rocker bottom shoes for the same insoles.
Paton et al. 2012 [50]	UK	RCT	18 months	119	Neuropathic diabetic foot ulceration	Prefabricated and custom-made insole	In-shoe pressure reduction, PTI, forefoot rate of load, total contact area	Prefab versus custom insoles, PPP ≥ 6%,
Praet et al. 2003 [51]	Netherlands	Repeated measure	Not reported	10	Diabetic Neuropathy No active ulcer, No major foot deformities	Three different types of footwear designs	Peak pressure reduction at multiple areas under the foot	Rocker sole can offload the forefoot area by 65%
Preece et al. 2017 [52]	UK	Case-control	Not reported	168	Diabetic Neuropathy (*n* = 17) Healthy control (*N* = 66)	Eight types of rocker sole design	Pressure reduction threshold of ≤200 kPa	Rocker apex position at 52%, 20^0^ rocker angle, 95^0^ apex angle yields effective offloading at most
Tang et al. 2014 [53]	Sweden	RCT	Two years	114	Diabetic neuropathy Angiopathy Foot deformities Previous ulcers or amputation	Three types of insoles, custom made (35 & 55° shore hardness EVA) vs prefab insoles with hardcore EVA + soft microfiber top cover (Control)	PPP PTI	The overall PPP for the insoles was between 180 kPa to 211 kPa, PTI differences 14 kPa/sec & 20 kPa/sec with Control.
Teffler et al. 2017 [54]	UK	Randomised crossover	Not reported	20	Diabetic neuropathy Increased forefoot plantar pressure No Charcot foot or partial amputation	Three types of insoles 1) Standard (Shape-based), milled insoles, 2) Milled, virtually optimised insoles and 3) 3D printed virtually optimised insoles	PPP	Virtually optimised insole reduced PPP by a mean of 41.3 kPa for milled and 40.5 kPa for 3D printed insoles in the same participants’ group.
Tsung et al. 2004 [55]	China	Case-control	Not reported	14	Diabetic neuropathy No Charcot foot or partial amputation Control: no foot deformity	Five support conditions including footwear-only, flat insoles; and three custom-made insoles with three weight-bearing conditions; 1) Full weight-bearing (FWB), 2) Semi-weight-bearing (SWB) and 3) Non-weight-bearing (NWB)	MPP PTI Mean contact area	For 2–3 MTH regions, SWB insoles yield maximum offloading comparing to two other insoles type. For MTH1, NWB insoles provide maximum offloading. FWB insoles show maximum PTI comparing to NWB & SWB conditions. NWB insoles provide maximum arch support and contoured shaped insoles.

**Table 2:** Study characteristics of selected articles for ulcer recurrence as the primary outcome measure

Author, date	Location	Study design	Follow up period	Sample size	Sample characteristics	Intervention & Comparison	Outcome measures	Result
Busch et al. 2003 [56]	Germany	Prospective cohort	Up to 42 months	92	Diabetes Neuropathy Peripheral vascular disease (PVD)	Lucro SDS vs non-SDS standard footwear	Ulcer recurrence	Annual ulcer recurrence SDS 15% vs Non-SDS 60% when severe foot deformity is non-existent
Bus et al. 2013 [57]	Netherlands	RCT	18 months	171	Diabetes Neuropathy Healed plantar ulcers	Custom-made footwear with and without modifications based on in-shoe pressure analysis	Ulcer recurrence Adherence of ≥80% steps taken	Modified custom-made footwear are only useful in offloading forefoot area if they are worn as per advised (Adherence ≥80%)
Chantelau et al. 1990 [58]	Germany	Prospective cohort	25 months	50	Diabetes Neuropathy PVD History of healed plantar foot ulcer Partial or forefoot amputation	Custom-made footwear with rocker soles and custom-made insoles with 10 mm thickness,	Ulcer recurrence Adherence (regular vs irregular wearing of footwear and insoles)	Regular wearing of footwear and insoles reduced the relative risk of foot ulceration to 0.48 (95% confidence interval 0.29 to 0.79), compared with irregular wearing
Lavery et al. 2012 [59]	USA	RCT	18 months	299	Diabetes Neuropathy Healed foot ulcers Foot deformity	Shear reducing insole (SRI) with standard therapy group (STG) with therapeutic footwear, diabetic foot education and care	Ulcer recurrence	SRI group were 3.5 times less likely to develop foot ulcers comparing to the STG group. No significant difference in the frequency of footwear and insole usage in SRI or STG group.
López-Moral et al. 2019 [60]	Italy	RCT	18 months	51	Diabetes Neuropathy Healed plantar ulcers	Semi-rigid (control) and rigid rocker sole (test) therapeutic footwear	Ulcer recurrence Adherence > 60%	Rigid rocker sole can reduce risk of re-ulceration at forefoot by 64% compared to semi-rigid rocker sole
Rizzo et al. 2012 [61]	Italy	RCT	5 years	298	Diabetes Neuropathy Healed plantar foot ulcer Minor amputation	Standard comfort footwear vs custom insoles and footwear as per Dahmen et al. algorithm	Ulcer recurrence	Ulcer recurrence rates in 1, 3 & 5 years are 11.5% vs 38.6, 17.6% vs 61, 23.5% vs 72% where forefoot deformities are predominant among the participants.
Ulbrecht et al. 2014 [62]	USA	RCT	15 months	150	Diabetes Neuropathy Healed plantar foot ulcer (MTHs) Increased barefoot plantar pressure	Control: Standard custom-made insoles from three different suppliers Experimental: Insoles made according to the protocol in Owings et al. 2008.	Ulcerative or non-ulcerative lesions at the plantar forefoot in MTHs regions	Foot shape and plantar pressure-based custom insoles provide superior offloading than insoles made from foot shape and clinical insights.

*MP* Metatarsal Pad, *MB* Metatarsal Bar, *MD* Metatarsal Dome, *SDS* Stock Diabetic Shoes, *MTH1* First Metatarsal Head, *FTI* Force Time Integral, *PTI* Pressure Time Integral, *MPP* Mean Peak Pressure, *TCI* Total Contact Insoles, *SRI* Shear Reducing Insoles, *STG* Standard Therapy Group

### Participants and settings

The participants were over 18 years of age, and the sample sizes ranged from 10 to 299. All participants in treatment groups had diabetes, and the majority had neuropathy. Participants had active or healed plantar foot ulcers, amputation, foot deformities, increased barefoot plantar pressure, or peripheral vascular disease. Most (88%) of the studies recruited participants from developed countries within high-risk foot clinics and 12% from developing countries [63]. Study duration ranged from a single session to 5 years.

### Intervention

Eleven studies [30, 38, 39, 51, 52, 56–58, 60, 61] used footwear and insoles as the intervention. Of these, three studies [38, 57, 61] used footwear which was manufactured according to a consensus-based algorithm proposed by Dahmen et al. [53]. One study [52] specifically examined footwear rocker sole profiles. High footwear upper design feature was investigated by one study [51], and it reported that higher upper increased contact area but did not improve pressure reduction at the forefoot area.

Fourteen studies [30, 38–40, 42, 44, 46, 48, 49, 51, 53, 58, 61, 62] reported on the prescribers, manufacturers and modifiers of the therapeutic footwear and insoles. The footwear prescribers reported in the studies were rehabilitation physicians [30, 38], diabetologist, podologist [61], podiatric physician [49]. The manufacturers for therapeutic footwear were orthopaedic shoe technicians [30, 38, 39, 51, 61], and orthopaedic shoemakers [40, 42, 58], where orthopaedic shoe technicians have similar training like certified pedorthists [30]. Reported insole manufacturers or modifiers were orthotic technician [53], pedorthist [44, 49], pedorthist or orthotist [46, 48, 49, 62].

Fourteen studies [40, 42, 44, 46–50, 53–55, 59, 62, 64] used insoles as a primary intervention in standardised or participant’s footwear. All studies reported on the type of footwear they used with varying descriptions of the design features and almost all studies reported on the description of insole design features used by the studies respectively, except Preece et al. [52]. Studies that are focused on the insole as a primary intervention has used prefabricated extra-depth footwear or regular retail footwear [40, 42, 44–50, 53–55, 62].

Insole features have been described by some studies [39, 41, 45, 47, 49, 50, 53, 54, 56, 59, 60, 62, 64] such as base, mid-layer, and top cover materials. The same authors also assessed hardness, thickness, casting and manufacturing technique, metatarsal dome or metatarsal bar, and arch support. Ten studies [40–42, 47, 48, 53, 55, 56, 59, 64] examined insole material thickness and hardness. Other components of insole configurations reported were application of metatarsal pad, metatarsal dome, or metatarsal bar [30, 39, 40, 42, 44, 46–48, 53, 57, 61] and their positioning [42, 44, 46–48, 53], arch support [30, 39, 40, 42, 51, 53, 55, 57, 61], top cover [30, 39, 42, 49–51, 53–57, 59, 61, 62, 64], adding local cushion to insole [39, 49, 57, 61, 62]. The size of the metatarsal dome or pad used by the studies is between 5 to 11 mm [42, 44, 47] in height, 66 to 74 mm in length, and 51 to 63 mm width [44]. The positioning of the metatarsal dome, bar or pad was between 5 to 10.6 mm proximal to MTHs [42, 44, 46] and at a line of 77% of PPP [47]. The size of extra arch support was 5 mm thick Lunalastic (NORA Freudenberg GmbH, Weinheim, Germany) in addition to arch support resulted from the casting technique [42]. Casting techniques for custom-insoles making, insole design, and manufacturing processes also have been reported by some studies [40, 47, 49, 54, 55, 62].

### Outcome measures

Eighteen studies [30, 38–42, 44, 46–55, 64] measured PPP as the primary outcome, and the majority measured this in-shoe. Most of the studies [30, 38, 39, 47, 50, 52, 53, 57, 64] used 200 kPa as an upper threshold to classify the intervention as successful offloading the foot. The remaining studies compared a baseline pressure assessment without the intervention to peak pressure reductions with the interventions. PTI and Force Time Integral (FTI) had also been assessed as a parallel outcome measure in some studies [40, 48–51, 53, 55]. Other studies [50, 51, 55, 64] also measured contact area and soft tissue thickness (STT) [46, 48] as a parallel outcome. Some single parameters measured by the studies were maximum force, contact area [64], and walking convenience [42]. One study [41] reported foot-sole hardness as an indicator and reduction in shore hardness value. Six studies [56–61] reported ulcer recurrence as a primary outcome measure and another study [62] reported on ulcerative and non-ulcerative lesions as the primary outcome. Three studies [57, 58, 60] measured patient adherence in their study as a secondary outcome.

The Pedar-X system (Novel GmbH, Germany) was the most commonly used in-shoe plantar pressure measuring device by studies [30, 38–40, 45, 47, 49, 50, 54, 57] followed by the F-Scan system (Tekscan Inc. USA) [42, 44, 46, 48, 53, 55]. Other systems included RS Scan system (RSScan, Ole, Belgium) [51]. Charanya et al. [41] used a pedobarograph system developed by Patil et al. [65–67] to capture the walking foot pressure image and data analysis.

The sensor’s thickness of the Pedar-X system is 2 mm [39, 40], F-Scan 0.18 mm [55], and RS Scan 0.7 mm [51]. Both sensors of Pedar-X and F-Scan collect pressure data at 50 Hz [44, 47], and both have four sensors per cm^2^ [38, 53]. RS Scan sensors collect data at 500 Hz [51]. Studies using Pedar-X systems used steps between 20 to 40 [40, 47, 49, 54] and 10 to 20 m walk-way [38, 45, 49]. Studies using F-Scan systems used walk-way length between 6.1 to 10 m [44, 55]. RS Scan collected dynamic in-shoe pressure data for 8 s (10–16 steps) [51].

### Reductions in forefoot plantar pressure

Arts et al. [38] reported on the effectiveness of footwear and insole design based on the algorithm proposed by Dahmen et al. [68]. The rate of pressure reduction was lower at the metatarsals area (29–50%) compared to midfoot (81%) and known ulcer location (62%) [38] when footwear and insoles are designed according to Dahmen’s algorithm.

Sole design (rocker sole) was the most reported design feature and some reported on detailed configurations such as rocker apex position [30, 38, 41, 50–52, 56, 61], rocker apex angle [52], rocker angle [30, 51, 52, 60], rigidity or hardness [30, 38, 41, 42, 53, 56, 60, 61] and, material type [41, 50, 51, 60, 61]. A rocker sole configuration with apex position at 52% of the footwear length, 20° rocker angle, and 95° apex angle can yield peak pressure < 200 kPa in 71–81% cases [52].

Some studies reported on footwear upper design features, such as upper height (high footwear 16 cm, Bottine 12.5 cm, Low footwear (6.5 cm) [38, 51, 61], footwear depth [39, 50, 56, 57, 60, 64], leg and tongue profile [38, 57, 61]. Other design features are; upper material, collar, lining, toe puff [50, 56, 60], heel counter, fastening system [53, 60] and active heel height [51].

Non-weight-bearing (NWB) casting technique yields more effective custom-made insoles to offload the hallux region and semi-weight-bearing (SWB) casting technique is more effective to offload 1–3 metatarsal heads (MTHs) [55]. The NWB insoles also yield the highest arch support comparing to insoles made by other casting techniques [55].

Insoles designed based on foot shape and plantar pressure data are more effective to offload the forefoot region compared to insoles designed based on foot shape only [49, 54, 62]. The outcome can be between 32 to 21% improvement from shape-only and traditionally manufactured insoles out of polypropylene base [49].

Custom-made insoles with multi-density, softer materials have demonstrated improved forefoot offloading compared to higher-density EVA (55° shore A). Extra arch support, metatarsal pads, a plastazote top cover, and local cushioning can further reduce plantar forefoot pressure [42, 64]. Metatarsal pad, local cushion and a plastazote top cover can reduce peak pressure by 14 to 15.9% on their own. A plastazote top cover combined with a metatarsal pad and local cushioning reduces 24 and 22% PPP at the forefoot [39].

### Reductions in ulcer recurrence

López-Moral et al. [60] explored the effect of two rocker soles: semi-rigid (Wellwalk technology with Vibram Strips) and rigid on the recurrence of ulceration. By using, a rigid rocker sole the risk of re-ulceration at the forefoot was reduced by 64% when compared with semi-rigid rocker sole footwear.

Busch et al. [56] examined the effect of two different footwear (Lucro stock diabetic footwear versus regular retail footwear) with insoles on ulcer relapse of 92 participants with high-risk neuropathic feet at 12 and 42 months. The footwear was available in three different widths with differing features: rocker bottom outsoles and soft upper with three layers. This combined footwear and insoles reduced ulcer relapse by 45% compared with standard footwear within the first year.

Rizzo et al. [61] compared a treatment group who were given therapeutic footwear designed as per Dahmen et al. [57, 68] and custom-made insoles to a control group who received standard footwear. The participants were assessed for ulcer occurrence and relapse at 12, 36 and 60 months. Ulcer relapse rates were significantly lower (11.5% versus 38.6% at 12 months, 17.6% versus 61% at 36 months and 23.5% versus 72% at 60 months) in the treatment group than controls.

Lavery et al. [59] examined the effect of shear-reducing insoles on ulcer recurrence when compared with standard insoles in the same style of footwear. Shear-reducing insoles were 3.5 times less likely to create ulcers in the study participants compared to the standard insoles, although, both insole types demonstrated equivalent plantar pressure reduction [69].

In another study [57] based on the algorithm proposed by Dahmen et al. [57, 68] the treatment group received custom-made footwear that was adjusted following in-shoe pressure analysis. Controls received custom-made footwear without the in-shoe pressure analysis. The primary outcome was ulcer relapse after 18 months. The outcomes were not significantly different due, in part, to variance in patient adherence.

## Discussion

Footwear and insoles are complex biomechanical interventions due to variance in design, materials, manufacturing methods, individual preferences and rates of adherence. This complexity is compounded when it is considered alongside the range of foot pathologies that co-exist with diabetes. Forefoot structural deformities are prevalent in this patient group [11, 12] increasing in-shoe plantar pressure at the metatarsal heads. The importance of footwear and insoles in offloading PPP for preventing plantar forefoot foot ulceration is well documented [70, 71]. However, the specifications of design parameters and materials that can reduce PPP at the forefoot area are not precise. Reduction of PPP is one of the major factors to reduce the risk of ulcer occurrence and recurrence. This review explores the identification of critical design features and materials used in footwear and insole manufacturing that can reduce PPP at the forefoot and prevent ulcer occurrence and recurrence. Summery of those features that are available in the literature has been presented in Appendix 1 and 2.

Several studies have suggested rocker sole profile as the most recommended design to offload PPP at the forefoot [30, 39, 51, 52, 56, 60, 61]. The studies showed strong evidence for the rocker sole with evidence pointing towards specific variations of the rocker sole: such as apex position, apex angle, rocker angle and rigidity of sole materials. An RCT [60] showed that a rocker sole configuration with the pivot point under the metatarsal heads and rigid sole materials improve plantar pressure offloading at the forefoot compared to rocker sole made with semi-rigid materials. In a 6 month follow-up, the plantar ulcer recurrence rate was 23 and 64% among the experimental and control group where sole rigidity was the only variant. Preece et al. [52] and Praet et al. [51] compared apex position and rocker angle for rocker sole design in their studies. They recommended an apex position at 52–63% of shoe length and rocker angle of 20–23^0^ to provide effective offloading at the forefoot (< 200 kPa), finding it more effective than any other lower or higher values of those respective parameters.

Arts et al. [38] in the Netherlands and Rizzo et al. [61] in Italy tested the effect of footwear design suggested by the consensus-based algorithm proposed by Dahmen et al. [68]. The key footwear design features in Dahmen algorithm are based on medical conditions. For example, the recommendations for a person with diabetes and history of neuropathic ulcers are footwear with a high upper (above ankle boots), stiffened tongue and leg uppers, rigid rocker soles with early pivot point. Both studies used above-ankle boots with custom-made insoles to offload pressure at the forefoot area. Both studies found that footwear and insoles designed according to this algorithm, are effective in offloading the neuropathic diabetic foot. However, Arts and colleagues [38] found that the algorithm is not as effective for footwear specifications to offload plantar pressure at the metatarsal heads.

There is a lack of guidance in the literature on footwear modifications that offload the forefoot. Footwear modification (also known as footwear customisation or optimisation) is common in both prefabricated and fully custom-made footwear. Most frequent footwear modifications are a re-configuration of rocker sole profile, such as early or significant pivot point (rocker angle) and stiffening the outer sole [30, 39]. Footwear modification success (≤200 kPa) is least at the forefoot [38, 39]. Bus et al. [30] recommended in-shoe plantar pressure analysis as an effective tool to guide the modifications for offloading the target regions in the neuropathic foot.

Insole modification features include local cushioning, replacing top covers with plastazote and applying a new or re-positioning existing metatarsal bars and metatarsal domes [30, 39, 47, 61], removing plugs, and adding arch supports [61, 64]. These are the most effective (PPP reduced ≤200 kPa) modifications in offloading or reducing PPP in targeted regions [30, 39]. The targeted regions were determined by the history of ulceration or from PPP measurements data. These modifications in the insole are proven to be effective in offloading plantar pressure at an optimal level. However, they are least effective in offloading pressure at the metatarsal heads [38, 39].

Pedorthists commonly use a higher upper height in their treatment of neuropathic forefoot ulcers. Dahmen et al. [68] and Diabetic Foot Australia (DFA) guideline [34] support such practice. However, Praet et al. [51] showed that high-ankle boots did not influence plantar pressure offloading when compared with low cut footwear. The authors suggest that although high-ankle boots do not change plantar pressures, they may reduce shear forces inside the shoe at the forefoot by increasing contact area around the ankle. Considering these findings, further studies assessing high-ankle boots will help to inform clinicians working in this field.

Many design features were not examined in the literature. Higher quality research is required to scientifically examine other important footwear design parameters, including heel height, toe height, upper materials, sole materials, heel counters, and closure systems for this therapeutic target.

There was moderate evidence [72] to suggest using total contact insoles [49, 55, 61, 62], metatarsal pads [40, 44, 46, 48, 62], metatarsal bars [47, 61] and plastazote top covers [39] to reduce PPP. Arts et al. [39] recommended plastazote as a top cover over leather due to its superiority in peak pressure offloading, but they need to be replaced every 6 months. Two studies [50, 53] also included prefabricated insoles as interventions, which also showed a reduction in forefoot plantar pressure.

In practice, the use of custom-made insoles over prefabricated devices needs to be considered in relation to cost versus benefit. Paton and colleagues [50] used two different insoles, made out of EVA and Poron, and compared cost as well. Custom devices were 18% higher cost in delivery than prefabricated insoles. The main difference was where the foot was cast to make the insoles, or insoles were selected from stock. There was no significant difference in PPP reduction between the two types of insoles. Custom-made insoles were, however, found to reduce PTI more than prefabricated insoles and lasted longer [50]. Customised devices may be preferred in practice as they account for structural changes in the diabetic foot, which is likely the reason that they reduce PTI more than prefabricated devices. Other studies [30, 40, 46, 48, 53, 55] that compared PPP reduction capacity of the custom-made insoles with prefabricated insoles and not examined the cost, those found custom-made insoles to be more effective in pressure offloading in almost every region of the foot.

Most common insole base materials are EVA with the hardness of 50–55^0^ Shore A and 30–35^0^ Shore A [47, 53] and the latter material showed improved performance in offloading PPP. However, the medium-density EVA base (30–35^0^ Shore A) insoles need more frequent replacement than the higher density EVA group insoles due to material fatigue.

PPT or Poron as mid-layer [56] and top cover materials either MCR, plastozote or microfiber are effective in plantar forefoot pressure offloading. PPT or Poron is also used as a top cover in some insole designs [56, 64]. Use of a leather top cover is of limited benefit due to its poor pressure reduction capacity [39].

None of the studies looked at the prevention of initial neuropathic plantar forefoot ulcer occurrence rather than a subsequent recurrence ulcer. Additionally, studies did not assess forefoot ulceration in isolation, but whole foot ulceration. PPP reduction in different regions requires different types of offloading. Further, different footwear and insole design features show differences in pressure reduction efficacy in different regions of the foot. The articles relied on in-shoe plantar pressure measurement data as a predictor of ulceration. However, other factors such as co-morbidity and lack of adherence to treatment also contribute to ulcer occurrence.

Plantar tissue stress incorporates vertical plantar pressure, horizontal shear pressure, and the frequency at which it is applied [73]. The reliance on plantar pressures as a predictor of ulceration may, therefore, be only one part of the picture. Lavery et al. [59, 69] reported that two different insoles (shear-reducing and standard insoles) with equivalent plantar pressure reduction capacity could have a significantly different outcome in ulcer recurrence where shear-reducing is the only differentiation factor. Shear-reducing insoles had 3.5 times higher ulcer prevention capacity than the standard insoles in the study participants. Since design features are likely to influence footwear function, and therefore, adherence, it is important to consider which features may prevent ulceration.

There is limited data in the literature to determine the efficacy of footwear in preventing ulcer occurrence. Preece et al. [52] and Martinez-Santos et al. [47] explored the efficacy of footwear and insole design features, but could not make any recommendations for preventing ulcer occurrence.

In this review, the articles were excluded if the participants had heel ulcer, Charcot foot or any active, dorsal foot ulcers, and these might limit the representation of complete diabetic foot conditions. This may limit the footwear and insole feature recommendations for those feet that have those conditions.

Heterogeneity in study designs, interventions, outcome measures and footwear and insoles design features make it also very difficult to come into a conclusion. Greater variations in participant’s inclusion criteria and foot deformities, footwear and insole types, their measuring, casting and designing techniques, in-shoe pressure analysis systems may result in inconsistent data. Hence, we can not make a clear comparison or pool data to analyse further.

Because of the need to customise to the individual, the success of custom-made footwear as an intervention in offloading the plantar foot is dependent on the knowledge and skills of the prescribers and manufacturers [30, 40, 55]. The studies in this review used a variety of skilled practitioners in these roles such as orthopaedic shoemakers, pedorthists depending on the region. The presence of these practitioners in the interdisciplinary team approach in high-risk foot services is increasingly recognised ([34], http://nadc.net.au/foot-network/).

Several studies [30, 42, 50, 57, 60, 61] explored patient satisfaction and adherence to wearing footwear and insoles. Patient adherence to wearing therapeutic footwear is vital to ensure improved offloading and ulcer prevention [57, 60, 61]. No difference was found in patients’ perceptions of custom-made versus prefabricated insoles [50]. Adding arch support and large metatarsal domes to basic insoles reduces patient adherence and walking comfort, despite evidence that these features improve pressure offloading [42].

Studies did not report the factors that influence adherence to therapy, which also limits the application of our findings. Consideration of patient expectations, effective education on footwear and activity-specific device designs are limited in the literature. Studies also did not consider geographical and socioeconomic factors. Most studies [30, 38, 39, 42, 47, 48, 50–53, 56, 57, 60, 61] were carried out in developed countries [63] with climates conducive to using ankle-high boots. Also, the practicality of these ankle-high boots for countries with warmer climates needs revisiting concerning patient adherence.

There was no study to take a personalised-treatment approach to focus on an individual’s need or preference to increase adherence. Footwear is a very personal item, and a pre-study participant’s feedback on their future footwear is crucial as opposed to only post-study feedback as adherence plays a vital role in an individual’s outcome [51, 57, 58, 60]. Study designs like the N-of-1 or single-patient-trial design [74, 75] may bridge the gap in the literature.

Appropriate footwear design that takes into consideration the needs of low-income countries and those with warmer climates are limited in the literature, even though the prevalence of diabetes tend to be higher among the populations in these regions [76].

## Conclusion

There is limited evidence to inform footwear and insole interventions, especially in conjunction with in-shoe plantar pressure reduction. The available evidence supports the identification of footwear and insole design and modification parameters that can influence forefoot plantar pressure reduction. Prevention of ulcer occurrence or recurrence at the plantar forefoot region in diabetic patients is limited. Further research is needed to improve care for people with diabetic foot ulceration.

## Supplementary information

**Additional file 1.** Search term and strategy.

**Additional file 2.** Quality assessment of the included articles.

## Data Availability

The author can be contacted for any data requests.

## Appendix 1

**Table 3:** Description of footwear features designed to reduce neuropathic forefoot plantar ulcer occurrence found in the literature

The description provided on footwear upper and sole design	Study(s)
Bottine (12.5 cm) or high footwear (16 cm) for upper height The toughened outsole, resilient material on the heel Toughened leg and tongue Rocker profile outsole with early and normal pivot point	Arts et al., 2012 [38], Bus et al. 2013 [57] Preece et al. 2017 [52] Rizzo et al. 2012 [61]
Fully custom-made orthopaedic footwear and semi-custom (extra depth + width off-the-shelf footwear) Thin, seamless cotton socks	Arts et al. 2015 [39]
Lucro stock diabetic footwear (SDS) with toughened outer-sole with forefoot rocker	Busch et al. 2003 [56]
Fully custom footwear manufactured with features of Ankle-high footwear, stiffened rubber outsole with rocker bottom sole. Modification: Outsole rocker pivot point relocation and rocker angle	Bus et al. 2011 [30]
Toughened rocker profile rubber outsole, shoes or sandals with smooth leather, adjustable front and back straps for sandals or closed in footwear	Charanya et al. 2004 [41]
Van Lier®, Netherlands, Outer sole shore type A: 86	Guldemond et al. 2007 [42]
Standard diabetic footwear (extra depth leather footwear, Dr. Foot Technology Co.,)	Lin et al. 2013 [64]
Semi-rigid rocker sole (Wellwalk technology with Vibram Strips) and rigid rocker sole (reinforced with composite fibre). The rocker sole was anteroposterior rocker and pivot point behind the metatarsal heads with 20 ° rocker angle. The shoes had rigid heel counter, extra depth toe boxes (14 to 16 mm deeper than standard shoes), lace or buckle closures.	López-Moral et al. 2019 [60]
SoleTech new footwear, style E3010	Mueller et al. 2006 [48]
Modular non-bespoke diabetic footwear with soft leather upper, plain vamp, secure fastening, microfibre lining material, padded collar, wall toe puff, EVA micro rubber sole unit with rocker where the apex is posterior to metatarsophalangeal joints line (County Orthopedic Footwear Ltd).	Paton et al. 2012 [50]
Eight types of rocker sole configuration by two types of rocker angle 15^0^ & 20^0^ each for the apex positions of 52, 57, 62, 67% of footwear length. (Duna, Italy)	Preece et al. 2017 [52]
Semi-rigid outer sole or stiff rocker sole, a stable heel counter, and adjustable laces or Velcro straps	Tang et al. 2014 [53]
Custom-made insoles crafted for each individual foot	Arts et al., 2012 [38]
Most frequent single modifications are replacement top cover of the insole, local cushioning of the insole, the addition of pad to the insole. Combined modification of insole: Above items and removal of local materials as an addition.	Arts et al. 2015 [39]
Flat insoles with rear base: 42^0^ Shore hardness and anterior base 20^0^ Shore hardness 6 mm thick Lunasoft® and 3 mm overall top-cover of PPT with 17^0^ Shore A hardness.	Busch et al. 2003 [56]

## Appendix 2

**Table 4:** Description of insole features designed to reduce neuropathic forefoot plantar ulcer occurrence found in the literature

The description provided on insole design	Study(s)
Fully custom-made insoles with multi-density and multi-layered materials, an open-cell or cross cell material top cover. Modification: Local removal of material on the insole, local softening, adding metatarsal, hallux pad or bar on the insole, replacement of the top cover	Bus et al. 2011 [30]
Custom made insole made from multilayered materials with cork base added with micro cork, a mid-layer of EVA base multiform. Additional metatarsal pad or bar with extra arch support.	Bus et al. 2013 [57]
Insole made of 12 mm microcellular rubber (MCR), shore value 20^0^	Charanya et al. 2004 [41]
Metatarsal dome, arch supports, and extra arch supports Insoles made of 5 mm Lunalastic as the top layer, 8 mm Lunasoft SL as the bottom layer, 1.1 mm Rhenoflex 3208® as internal reinforcement. Every layer of arch support has 5 mm thickness of Lunalastic material.	Guldemond et al. 2007 [42]
3 mm Shore A 35^0^ EVA as 1st layer, 2 mm Velcro and velvet in 2nd layer and 6 mm Shore A 50^0^ Poron in the third layer	Lin et al. 2013 [64]
Multilayered with 40° shore hardness EVA base and Poron top cover, cut-out in the affected metatarsal head.	López-Moral et al. 2019 [60]
Insole base with 5 mm 50^0^ Shore A EVA with three different metatarsal bar (MB) positioning out of two different types materials: 20^0^ Shore A EVA, 20^0^ Shore A Poron	Martinez-Santos et al. 2019 [47]
1.27 cm thick number 2 plastazote with shore value approx. 35, metatarsal pad (MP), positioned proximal to metatarsal heads	Mueller et al. 2006 [48]
Full length 3 mm blue medium density Ethylene Vinyl Acetate (EVA) shell and 6 mm grey Poron top cover	Paton et al. 2012 [50]
SM-2, 3 & 4: ¾ custom insoles with EVA base and 3 mm PPT full-length top cover SM-5 & 6: Custom insoles with EVA base and 3 mm PPT full-length top cover	Praet et al. 2003 [51]
Insoles from the static footprint and foam box impression, configured with arch support, metatarsal bar, soft fillers. Insole materials: PPT, Duuroterm, Alcaform	Rizzo et al. 2012 [61]
Custom insoles: 35 & 55^0^ Shore A hardness EVA (14 mm thickness) for custom made insoles manufactured from positive plaster moulds, metatarsal bars proximal to II-IV MTH’s. Prefabricated insole: Hardcore EVA base, 12^0^ Shore hardness microfiber top layer (GloboTec® comfort 312,750,501,400)	Tang et al. 2014 [53]

## Abbreviations

RCT: Randomised controlled trial
PRISMA: Preferred reporting items for systematic reviews and meta-analysis
BMI: Body mass index
DPN: Diabetic peripheral neuropathy
PPP: Peak plantar pressure
PTI: Pressure time integral
STT: Soft tissue thickness
DFA: Diabetic foot Australia
HRFS: High-risk foot services
