# Supplementary material for: Footwear and insole design features that reduce neuropathic plantar forefoot ulcer risk in people with diabetes: a systematic literature review
Source: J Foot Ankle Res. 2020 Jun 4;13:30. doi: 10.1186/s13047-020-00400-4 (PMC7271493; doi:10.1186/s13047-020-00400-4)
Supplement: Supplementary file 1 — Additional file 1. Search term and strategy. [file 13047_2020_400_MOESM1_ESM.docx]

### Additional file 1 Database and search terms used to identify literature for review

| **Database** | **Search terms** | **Citations reviewed** |
| --- | --- | --- |
| **Medline** | ((TOPIC: ((("Diabetic foot") "Diabetic foot" OR "physio pathology") OR "foot deformities") OR "Neuropath*") AND TOPIC: (((((("Footwear") OR "Orthoses") OR "Shoe") OR "footwear prescription") OR "insole") OR "innersole") OR "sock*")) AND TOPIC: ((("Ulcer prevention") OR "Offloading") OR "Foot Ulcer") OR "Plantar pressure")) | 238 |
| Limiters | 1987-2019 [Published date]; Full text; English [Language] |  |
| **Cinahl** | TX ( ("Diabetic foot" OR "physio pathology" OR "foot deformities" OR "Neuropath*") ) AND TX ( ("Footwear" OR "Orthoses" OR "Shoe" OR "footwear prescription" OR "insole" OR "innersole" OR "sock*") ) AND TX ( ("Ulcer prevention" OR "Offloading" OR "Foot Ulcer" OR "Plantar pressure") ) | 201 |
| Limiters | 1987-2019 [Published date]; Full Text; English [Language]; Peer Reviewed; Research Article [Document type]; Research [Publication Type] |  |
| **ProQuest** | FT(("Diabetic foot" OR "physic pathology" OR "foot deformities" OR "Neuropath*") AND ("Footwear" OR "orthoses" OR "Shoe" OR "footwear prescription" OR "insole" OR "innersole" OR "sock*") AND ("Ulcer prevention" OR "offloading" OR "Foot Ulcer" OR "Plantar pressure")) | 971 |
| Limiters | 1987-2019 [Published date]; Scholarly Journals [Source type]; Article [Document type]; English [Language] |  |

### Additional file 1 Database and search terms used to identify literature for review (Continued)

| **Database** | **Search terms** | **Citations reviewed** |
| --- | --- | --- |
| **Amed** | TX ( ("Diabetic foot" OR "physio pathology" OR "foot deformities" OR "Neuropath*") ) AND TX ( ("Footwear" OR "Orthoses" OR "Shoe" OR "footwear prescription" OR "insole" OR "innersole" OR "sock*") ) AND TX ( ("Ulcer prevention" OR "Offloading" OR "Foot Ulcer" OR "Plantar pressure") ) | 91 |
| Limiters | 1987-2019 [Published date]; English [Language] |  |
| **Scopus** | TITLE-ABS-KEY(("Diabetic foot" OR "physio pathology" OR "foot deformities" OR "Neuropath*") AND ("Footwear" OR "Orthoses" OR "Shoe" OR "footwear prescription" OR "insole" OR "innersole" OR "sock*") AND ("Ulcer prevention" OR "Offloading" OR "Foot Ulcer" OR "Plantar p*")) | 639 |
| Limiters | 1987-2019 [Published date]; Article [Document type]; English [Language] |  |
| **Academic Search Premier** | TX ( ("Diabetic foot" OR "physio pathology" OR "foot deformities" OR "Neuropath*") ) AND TX ( ("Footwear" OR "Orthoses" OR "Shoe" OR "footwear prescription" OR "insole" OR "innersole" OR "sock*") ) AND TX ( ("Ulcer prevention" OR "Offloading" OR "Foot Ulcer" OR "Plantar pressure") ) | 410 |
| Limiters | 1987-2019 [Published date]; Scholarly (Peer Reviewed) Journals [Document type]; Full text; English [Language] |  |
| Total records | | 2,550 |
| Total records after duplicates removed | | 1,771 |
