# Supplementary material for: Footwear and insole design features that reduce neuropathic plantar forefoot ulcer risk in people with diabetes: a systematic literature review
Source: J Foot Ankle Res. 2020 Jun 4;13:30. doi: 10.1186/s13047-020-00400-4 (PMC7271493; doi:10.1186/s13047-020-00400-4)
Supplement: Supplementary file 2 — Additional file 2. Quality assessment of the included articles. [file 13047_2020_400_MOESM2_ESM.docx]

Additional file 2. Quantitative studies: McMaster Critical Review Form – Quantitative Studies*

|  | Arts et al. (2012) | Arts et al. (2015) | Bus et al. 2004 | Bus et al. (2011) | Bus et al. (2013) | Busch et al. (2003) | Chantelau et al. (1990) | Charanya et al. (2004) | Guldemond et al. (2007) | Hastings et al. (2007) | Lavery et al. (2012) | Lin et al. (2013) | López-Moral et al. (2019) | Lott et al. (2006) | Martinez-Santos et al. (2019) | Mueller et al. (2006) | Owings et al. (2008) | Paton et al. (2012) | Praet et al. (2003) | Preece et al. (2017) | Rizzo et al. (2012) | Tang et al. (2014) | Telfer et al. (2017) | Tsung et al. (2004) | Ulbrecht et al. (2014) |
| --- | --- | --- | --- | --- | --- | --- | --- | --- | --- | --- | --- | --- | --- | --- | --- | --- | --- | --- | --- | --- | --- | --- | --- | --- | --- |
| **Study Purpose**  Was the purpose stated clearly? | √ | √ | √ | √ | √ | √ | √ | √ | √ | √ | √ | √ | √ | √ | √ | √ |  | √ | √ | √ | √ | √ | √ | √ | √ |
| **Literature**  Was relevant background literature reviewed? | √ | √ | √ | √ | √ | √ | √ | √ | √ | √ | √ | √ | √ | √ | √ | √ |  | √ | √ | √ | √ | √ | √ | √ | √ |
| **Sample**  Was the sample described in detail?  Was the sample size justified? | √  X | √  X | √  X | √  X | √  √ | √  X | X  X | X  X | √  X | √  X | √  X | √  X | √  √ | √  X | √  √ | √  X |  | √  √ | √  X | √  X | √ X | √  √ | √  √ | √  √ | √  √ |

Additional file 2. Quantitative studies: McMaster Critical Review Form – Quantitative Studies* (Continued)

|  | Arts et al. (2012) | Arts et al. (2015) | Bus et al. 2004 | Bus et al. (2011) | Bus et al. (2013) | Busch et al. (2003) | Chantelau et al. (1990) | Charanya et al. (2004) | Guldemond et al. (2007) | Hastings et al. (2007) | Lavery et al. (2012) | Lin et al. (2013) | López-Moral et al. (2019) | Lott et al. (2006) | Martinez-Santos et al. (2019) | Mueller et al. (2006) | Owings et al. (2008) | Paton et al. (2012) | Praet et al. (2003) | Preece et al. (2017) | Rizzo et al. (2012) | Tang et al. (2014) | Telfer et al. (2017) | Tsung et al. (2004) | Ulbrecht et al. (2014) |
| --- | --- | --- | --- | --- | --- | --- | --- | --- | --- | --- | --- | --- | --- | --- | --- | --- | --- | --- | --- | --- | --- | --- | --- | --- | --- |
| **Outcomes**  Were the outcome measures reliable?  Were the outcome measures valid? | √  √ | √  √ | √  √ | √  NR | √  √ | NR  NR | √ | NR?X  NR?X | NR  NR | √  √ | √  √ | √  √ | √  √ | √  √ | √  √ | √  √ | √  √ | √  √ | √  √ | ?  ? | √ √ | √  √ | √  √ | √  √ | √  √ |
| **Intervention**  Was the intervention described in detail?  Was contamination avoided?  Co-intervention was avoided? | √  NA  NA | √  NA  NA | √  √  NA | √  NA  NA | √  √  NR | √  NR  √ | X  X  X | √  NR  NR | √  NA  √ | √  √  NA | X  X  X | √  √  √ | √  √  NA | √  X  √ | √  √  NA | √  NA  NA | √  NA  NA | √  √  √ | √  NA  NA | √  NA  √ | √ NA  √ | √  √  √ | √  √  NA | √  √  NA | √  √  NR |

Additional file 2. Quantitative studies: McMaster Critical Review Form – Quantitative Studies* (Continued)

|  | Arts et al. (2012) | Arts et al. (2015) | Bus et al. 2004 | Bus et al. (2011) | Bus et al. (2013) | Busch et al. (2003) | Chantelau et al. (1990) | Charanya et al. (2004) | Guldemond et al. (2007) | Hastings et al. (2007) | Lavery et al. (2012) | Lin et al. (2013) | López-Moral et al. (2019) | Lott et al. (2006) | Martinez-Santos et al. (2019) | Mueller et al. (2006) | Owings et al. (2008) | Paton et al. (2012) | Praet et al. (2003) | Preece et al. (2017) | Rizzo et al. (2012) | Tang et al. (2014) | Telfer et al. (2017) | Tsung et al. (2004) | Ulbrecht et al. (2014) |
| --- | --- | --- | --- | --- | --- | --- | --- | --- | --- | --- | --- | --- | --- | --- | --- | --- | --- | --- | --- | --- | --- | --- | --- | --- | --- |
| **Results**  Were results reported in terms of statistical significance?  Were the analysis method(s) appropriate?  Was the clinical importance reported?  Were drop-outs reported? | √  √  √  X/  NA | √  √  √  √ | √  √  √  √ | √  √  √  X/NA | √  √  √  √ | √  √  √  √ | √  √  √  X/NA | √  √  √  X | √  √  √  NA | √  √  √  √ | √  √  √  X/NA | √  √  √  X/NA | √  √  √  √ | √  √  √  X/NA | √  √  √  √ | √  √  √  X/NA | √  √  √  √ | √  √  √  √ | √  √  √  NA | √  √  √  NA | √  √  √  NA | √  √  √  X/NA | √  √  √  NA | √  √  √  X/NA | √  √  √  NA |
| **Conclusions and implications**  Conclusions were appropriate given study methods and results | √ | √ | √ | √ | √ | √ | √ | √ | √ | √ | √ | √ | √ | √ | √ | √ | √ | √ | √ | √ | √ | √ | √ | √ | √ |

**Notes**: *Only the key questions on the left-hand side have been reported, without question regarding study design as this is covered in data extraction; √ refers to criteria met within-study and × refers to criteria not met.

**Abbreviations**: **NR**, not reported; **NA**, not applicable.
